# Learners with Low Working Memory Capacity Benefit More from the Presence of an Instructor’s Face in Video Lectures

**DOI:** 10.3390/jintelligence11010005

**Published:** 2022-12-27

**Authors:** Yuyang Zhang, Jing Yang, Zhisheng (Edward) Wen

**Affiliations:** 1 Bilingual Cognition and Development Lab, Center for Linguistics and Applied Linguistics, Guangdong University of Foreign Studies, Guangzhou 510420, China; 2 Faculty of English Language and Culture, Guangdong University of Foreign Studies, Guangzhou 510420, China; 3 School of International Studies, Zhejiang University, Hangzhou 310058, China; 4 Faculty of Languages and Translation, Macao Polytechnic University, Macao SAR 999078, China

**Keywords:** video lectures, instructor presence, working memory, social presence, cognitive load, eye-tracking

## Abstract

This current study explores the influence of learners’ working memory capacity (WMC) on the facilitation effect of an instructor’s presence during video lectures. Sixty-four undergraduates were classified into high and low WMC groups based on their performance in an operation span task. They watched three types of video lectures on unfamiliar topics in a random order: video lectures with an instructor’s voiceover but without presence (VN), video lectures with the instructor’s face picture (VP), and video lectures with the same instructor talking (VV). We collected their eye movement data during the video lectures and their learning performance in the comprehension tests following each video. Two-way ANOVA and post-hoc analyses showed that the instructor’s presence significantly improved comprehension performance in only the low WMC group. They allocated more attention to the instructor’s face picture and talking head than the high WMC group. Our results highlight the value of the instructor’s presence as a social cue in video lectures, which is particularly beneficial for learners with a low WMC.

## 1. Introduction

As lecture videos become popular, research on the effectiveness of video design is expanding. An instructor’s presence in a video, as one of the most significant features of effective video design, has received increasing attention in recent years. Some studies demonstrated that the instructor’s presence promotes learning and induces positive affective arousal (e.g., Chen and Wu 2015; Colliot and Jamet 2018; Pi and Hong 2016; Van Gog et al. 2014; Wang and Antonenko 2017; Zhang and Yang 2022). As Mayer (2014, 2021) suggested, the onscreen instructor could act as a social cue that facilitates generative processing in multimedia learning. As a result, students actively interpret the learning materials and integrate new stimuli with their prior knowledge, thus improving their learning. 

However, a few studies showed that the instructor’s presence might distract and overburden the learners (e.g., Homer et al. 2008; Van Wermeskerken and Van Gog 2017; Van Wermeskerken et al. 2018; Wilson et al. 2018). Learners’ limited working memory capacity (WMC) would be overloaded by extraneous items or processes, according to the cognitive load theory (Sweller et al. 2011) and the cognitive theory of multimedia learning (Mayer 2021). Thus, the instructor’s presence in the video lecture, if not vital for the learning, may lead to the split-attention effect (Ayres and Sweller 2014; Sweller et al. 2011) and hinder learning.

So far, most research on the instructor presence effect has focused on the effectiveness of the video lecture design (i.e., the instructor’s eye gaze, body orientations, and gestures) (e.g., Pi et al. 2019, 2020, 2022). Only a few studies have examined the influence of the learners’ cognitive abilities on the effectiveness of video lecture design (e.g., the instructor’s presence in video lectures). For example, working memory (WM) refers to the limited capacity to handle cognitive demands in the service of mental tasks (Baddeley and Hitch 1974; Baddeley 2003; Sepp et al. 2019; Cowan 2022; Wen et al. 2022). The instructor’s presence may overload learners with a low WMC and impede learning, while this may not be the case for those with a high WMC. The present study, using the eye-tracking technique, aimed to investigate the potential influence of the instructor’s presence in video lectures on learners with a high or low WMC. 

### 1.1. Instructor Presence Effect and Working Memory Capacity

Instructional video design should guide learners’ cognitive processes to integrate new information with their existing knowledge without overloading their limited WMC (e.g., Sweller et al. 2011; Mayer 2021). Learners engage in three kinds of cognitive processing during multimedia learning, all drawing on their limited WMC (Sweller et al. 2011; Mayer 2021; DeLeeuw and Mayer 2008). First, extraneous cognitive processing refers to the cognitive processes that result from poor instructional designs and do not directly serve the instructional objectives. Second, essential cognitive processing, induced by the intrinsic complexity of the to-be-learned material, is required to represent the presented material in WM. Third, generative processing serves to make sense of the provided content and depends on the learners’ learning motivation. According to this triadic theory of multimedia learning (Mayer 2011), the general principles of a well-designed video lecture should aim to (a) eliminate extraneous processing, (b) manage essential processing, and (c) promote generative processing (Mayer 2021).

Online video learning lacks direct student-instructor interactions, compared with traditional face-to-face classroom instruction. Is the instructor’s presence in video lectures a distraction or an aid for learners with limited WMC? The social agency theory (Mayer 2014) postulates that positive social cues, such as the instructor’s presence, enhance the learners’ feelings of social partnership with the instructor. The instructor’s voice, eye gaze, facial expressions, and gestures may foster generative processing (Mayer 2021). As a result, learners actively engage in cognitive processing, leading to improved essential processing and learning outcomes. However, according to the embodiment principle (Mayer 2014, 2021), only a highly-embodied onscreen instructor serves as a positive social cue that primes the sense of the social stance in the learners. Learners may benefit little from the instructor’s picture as it is a low-embodiment cue (Moreno et al. 2001). 

Although the instructor’s presence may foster generative processing, it could also increase extraneous processing and leave less cognitive capacity for essential processing. Further, the instructor’s onscreen presence might distract learners, causing their attention to be constantly shifted from the content to the instructor and then back again. Frequent attention-switching will overload learners (Sweller et al. 2011; Sweller et al. 2022) and may lead to the split-attention effect (Ayres and Sweller 2014; Sweller et al. 2011). Suppose too much attention is given to the instructor at the expense of insufficient attention allocated to the learning content. In that case, the benefits of an onscreen instructor may be countered and potentially hinder learning (in the worst-case scenario).

Similarly to the conflicting theoretical propositions, the empirical evidence for the instructor presence effect is also mixed. As mentioned earlier, many studies have shown that the instructor’s presence in video lectures improves students’ learning performance (e.g., Kokoç et al. 2020; Mayer and DaPra 2012; Pi and Hong 2016; Zhang and Yang 2022; Wang et al. 2018) and affective responses (Wang and Antonenko 2017; Wang et al. 2020; for reviews, see Alemdag 2022; Henderson and Schroeder 2021). Others, however, have failed to replicate the facilitative effect of the instructor’s presence on learning outcomes (e.g., Homer et al. 2008, Van Wermeskerken and Van Gog 2017; Van Wermeskerken et al. 2018; Wilson et al. 2018). Instead, they found that the instructor’s presence attracted much of the learners’ visual attention (e.g., Van Wermeskerken and Van Gog 2017; Van Wermeskerken et al. 2018). For example, Homer et al. (2008) showed that participants self-reported having experienced higher cognitive loads while watching instructor-present videos rather than instructor-absent ones. That said, there were no significant differences in the learning performance among the video conditions. Interestingly, Wilson et al. (2018) reported that while the instructor’s presence impaired the learners’ comprehension performance, the learners preferred and believed that they learned most effectively in the video lectures with the instructor’s presence.

Variations in video lectures may cause contradictory results in existing research on the instructor presence effect: duration, learning domain, human embodiment, and study settings (Wang and Antonenko 2017; Hong et al. 2018; Pi et al. 2017; Yi et al. 2019; Zhang et al. 2021; for review, see Alemdag 2022). While numerous research focused on the design details of an instructor’s presence in video lectures, few studies have explored the influence of learners’ cognitive abilities on the instructor presence effect (Kokoç et al. 2020; Zhang and Yang 2022). Kokoç et al. (2020) investigated learners’ sustained attention and the interaction with the instructor’s presence during video lectures. In their study, individuals with high-sustained attention focused less on the lecturer’s image and benefited more from the instructor-present videos than the low-sustained attention group. The instructor presence effect is related to generative and extraneous processing, both constrained by learners’ WMC (Mayer 2021). Nevertheless, to our knowledge, no previous study has explored learners’ WMC and the instructor presence effect in video lectures. 

The limited WMC has been postulated as a key tenet of the cognitive load theory (Sweller et al. 2011; Sweller et al. 2022) and the triadic multimedia learning model (Mayer 2011, 2014). As a cognitive resource, WM is limited in terms of duration and capacity (Oberauer et al. 2018). During video lectures, students actively engage in multiple complex cognitive processes: they attend to the available information from multiple sources, select relevant information, and organize and integrate the presented information into memory. These multiple processes place differential demands on their limited WMC. As individuals differ in their WMC (Conway et al. 2007) or WM span (Conway et al. 2005), learners with different WMCs might differ in multimedia learning outcomes. Studies have shown that learners’ WMC predicts multimedia learning performance (a) when some appealing information is presented (Kam et al. 2020; Sanchez and Wiley 2006), (b) when the learning process is challenging to manage due to the presentation (Banas and Sanchez 2012; Lusk et al. 2009; Sanchez and Wiley 2009), or (c) when learners’ certain emotional states are activated (Knörzer et al. 2016). These studies suggest that learners with a higher WMC are less likely to be distracted by enticing details. They are better equipped to learn even in segmented presentations and are less influenced by their respective emotional states than those low-WMC learners. If the onscreen instructor’s presence increases extraneous processing, it might cause distinct influences on learners with different WMCs. However, no study has examined the interaction between learners’ WMC and the instructor’s presence effect in video lectures. The current study investigates the impact of learners’ WMC on the instructor presence effect in video lectures. 

### 1.2. Using Eye-Tracking to Explore Learning Process in Instructor-Present Video Lectures

The use of eye-tracking technology in learning research (Lai et al. 2013), particularly multimedia learning research (Alemdag and Cagiltay 2018), is gaining popularity. As a learning process measure, eye-tracking provides “an online protocol allowing the study of attention processes” (p. 414) and inferences about the cognitive activities involved in multimedia learning, including selecting, organizing, and integrating information (Alemdag and Cagiltay 2018). In their review, Alemdag and Cagiltay (2018) summarized the moderators of eye-tracking measurements in multimedia learning, which include individual differences. For example, Sanchez and Wiley (2006) demonstrated that learners of various WMCs differed in their comprehension scores and eye movement patterns when irrelevant images accompanied the text. In particular, learners with a high WMC spent less time looking at irrelevant images and eventually scored higher than those with a low WMC. The authors ascribed the high WMC group’s better performance to their better attention control ability.

An increasing number of researchers have utilized eye-tracking to investigate the instructor presence effect (e.g., Kokoç et al. 2020; Pi and Hong 2016; Van Wermeskerken et al. 2018; Wang and Antonenko 2017; Wang et al. 2020; Zhang and Yang 2022). Using eye metrics, such as fixation counts and dwell time, these studies revealed how learners allocated their attention when watching video lectures with or without an instructor present. In particular, they demonstrated that the onscreen instructor captured much of the learners’ attention as they tended to dwell less on the learning content in the instructor-present video than in the instructor-absent one. For instance, participants in Van Wermeskerken et al. (2018) spent around 30% of their time watching the instructor in the instructor-present condition. 

Despite the growing number of studies using the eye-tracking technique to explore the learning process of multimedia learning, little is known about the modulation of learners’ WMC in terms of their eye movements during the learning process (i.e., attention allocation). The current eye-tracking study explores the influence of an instructor’s presence on learners’ visual attention allocation in video lectures.

### 1.3. The Present Study

The current study examined the modulation of learners’ WMC on their learning process and learning performance when watching instructor-present/instructor-absent video lectures. For the vast majority of video-conferencing platforms, such as Zoom meetings, online instructors can easily switch on their cameras to be continuously present throughout lectures. In the current study, we presented all participants with three types of video formats: video lectures with an instructor’s narration but without their presence (VN), video lectures with the instructor’s neutral face image (a static headshot) (VP), and video lectures with the same instructor’s talking head (VV) embedded to explain the content in a synchronized manner. Each participant watched three videos in each of the three conditions (VN, VP, and VV), and their eye movements were recorded simultaneously. A comprehension test followed each video to assess participants’ learning performance. Since WM is typically measured by complex memory span tasks (Conway et al. 2005; Wen et al. 2021), we used an automated operation span test to evaluate participants’ WMC. A mean-split approach was adopted for classifying the participants into high and low WMC groups based on their operation span scores.

We hypothesized that the instructor presence effect would interact with individual differences in WMC to affect participants’ learning outcomes and attention allocation. Specifically, we expected learners with a high WMC would perform better in instructor-present videos than those with a low WMC. The low-WMC learners might allocate more attention to the presented instructor than their high-WMC counterparts. 

## 2. Materials and Methods 

### 2.1. Research Design 

This study adopted a three-by-two factorial mixed design, with video type (video- instructor’s talking video, VV; video-instructor’s face picture, VP; video–no instructor, VN) as a within-subjects variable and WMC (high WMC and low WMC) as a between-subjects variable. Unlike previous studies that usually set the video type as a between-subjects variable, this study adopted a within-subject design to eliminate the subject variance among video formats. All participants were requested to watch nine videos, with three in each of the three conditions. We randomized the presentation sequence of the nine videos across the participants to minimize fatigue and practice effects. Eye movement data were collected via an Eyelink 1000 eye tracker (SR Research Ltd., Kanata, ON, Canada) in the desktop-mounted mode. Participants sat approximately 60 cm from the screen and listened to the video via headphones connected to the computer. 

### 2.2. Participants

Sixty-four Chinese undergraduates^1^ (32 females and 32 males; mean age = 19.72 ± 1.02 years) participated in the current study. The participants were students majoring in English, translation, international business, and other disciplines. All participants had normal or corrected-to-normal vision and hearing. They provided written informed consent and received payment for their participation. The present study was approved by the ethical committee of the Bilingual Cognition and Development Lab at the Guangdong University of Foreign Studies, China.

### 2.3. Materials

In the current study, all videos were developed by the authors of this study (for details, see Supplementary Materials, Table S1). The scripts covered different topics, including science, history, and literature, and were adapted from texts from the Chinese version of Wikipedia (https://zh.wikipedia.org/wiki/Wikipedia (accessed on 2 May 2021)). Each text comprised approximately 400 words. Twenty Chinese college students, who were not involved in the eye-tracking experiment, evaluated the familiarity of each topic on a five-point Likert scale from 1 (not familiar at all) to 5 (very familiar). They also rated the difficulty of its content using a five-point Likert scale from 1 (not difficult at all) to 5 (very difficult). Based on their self-reports, the topics were unfamiliar (mean rating scores = 2.03 ± 1.22) and not difficult (mean rating scores = 2.23 ± 0.93). 

**Figure 1:**
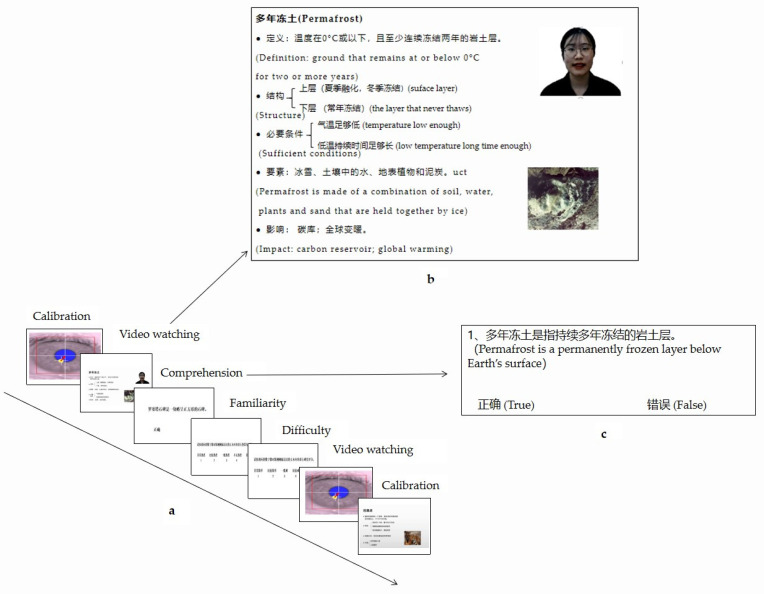
Eye-tracking experiment paradigm (**a**), a screenshot of a video in the video-instructor talking condition (VV) (**b**), and one sample of comprehension questions following the video (**c**).

Based on the scripts, the same instructor (a young female Chinese native speaker with a standard accent in Putonghua) recorded all of the videos in three different formats. In the VN condition, only the content (i.e., the written text and a topic-related picture) and the instructor’s narration were presented (as a voiceover in the background). In the VP condition, a picture of the instructor’s neutral face was presented in the upper-right corner—as the default talking head in Zoom meetings—along with the written text and a topic-related picture. In the VV condition, a video window of the instructor’s talking head was placed in the same location as the instructor’s picture in the VP condition (see Figure 1b). The visual texts and the auditory narration of the instructor were in Chinese, the learners’ first language. Each video lasted approximately two minutes. Two videos were displayed in the practice session, and nine in the eye-tracking experiment (see Table S1 in Supplementary Materials). Three videos on different topics were used for each condition to eliminate stimulus variances.

### 2.4. Measurements

#### 2.4.1. Comprehension Test

The comprehension test included eight true/false judgment questions following each video. The test assessed the participant’s retention of the content covered in the video. The comprehension questions would appear successively in the same window as the lecture video as soon as the video ended (Figure 1c). Participants were instructed to press the corresponding buttons (yes/no) to indicate true or false for each statement. Each correct answer received one point. Every participant completed 72 questions for all nine videos (the average accuracy was 85%). The maximum score they could receive in each video condition was 24.

#### 2.4.2. Familiarity and Difficulty Ratings

Following the comprehension test of each video, participants rated their familiarity with the video topic and the difficulty level of its content (using the same five-point Likert scales as in the pilot rating, see Supplementary Materials). The instruction clarified that familiarity referred to their prior knowledge of each topic. The participants involved in the eye-tracking experiment were unfamiliar with the topics (mean rating scores = 1.83 ± 0.91). They reported that the content of each topic was not difficult on the whole (mean rating scores = 2.54 ± 0.83).

#### 2.4.3. Visual Attention Allocation

We created three areas of interest (AOI) to investigate the learners’ visual attention distribution: the text, the topic-related picture, and the instructor. The size (the text area: 454 × 630 pixels; the topic-related picture area: 220 × 246 pixels; the instructor area: 260 × 260 pixels) and the location of each of the AOIs were the same across all video formats. In each AOI, we examined the participants’ fixation count percentage (average percentage of all fixations on a particular AOI) and dwell time percentage (average percentage of trial time spent on a particular AOI). Additionally, as in previous research (Wang and Antonenko 2017; Wang et al. 2020), we examined the number of transitions between the instructor area and the other two areas in the VP and VV conditions. Eye movement data from the videos of the same type were averaged for analysis. 

#### 2.4.4. WMC: An Automated Operation Span Test

We adopted the automated operation span test (Unsworth et al. 2005) to measure WMC. During the test, participants were presented with some simple math problems (e.g., 3 × 2  +  1  =  ?), with each followed by a number (e.g., 7) and then an English letter (e.g., F). They were asked to determine whether the answer to a math problem was the number presented (by clicking TRUE or FALSE). They were also asked to recall the letters by selecting letters in serial order from a matrix of 12 possible letters at the end of each series of math equations. This operation span test yielded five scores: Ospan score, total number correct, math errors, speed errors, and accuracy errors. Following Unsworth et al. (2005), we imposed an 85% accuracy criterion for all participants. 

We adopted the mean-split approach for grouping based on the Ospan scores. Thirty-two participants who scored higher than the mean score were included in the high WMC group, and 32 participants who scored lower than the mean were admitted to the low WMC group. An independent sample *t*-test showed that the high WMC group and the low WMC group differed significantly in their Ospan scores (M_high WMC_ = 63.84 ± 7.14, M_low WMC_ = 38.47 ± 9.33, *t*_62_ = 12.22, *p* < 0.001).

### 2.5. Procedure 

After signing the informed consent form, participants were tested individually in an eye-tracking room. A nine-point calibration procedure was applied prior to watching each video. Immediately after watching each video, participants were instructed to answer eight comprehension questions (by pressing the yes/no buttons on the keyboard). They then rated the familiarity and difficulty of each video (by pressing the keys on the keyboard, from 1 to 5). Participants watched the nine videos in a randomized order. Figure 1a presents a flowchart of the entire video-watching task procedure. After the eye-tracking experiment, participants were asked to complete the operation span test. The total duration of this study was approximately one hour.

## 3. Results

### 3.1. Effects of Video Type and WMC on Learning Performance

Table 1 summarizes each condition’s means and standard deviations for high and low WMC groups. Two-way ANOVA revealed a significant main effect of the instructor video type (F(2, 62) = 9.54, *p* < 0.001, η^2^ = 0.13) but not WMC (F(1, 62) = 1.87, *p* = 0.176, η^2^ = 0.029). However, the interaction between the video type and WMC was significant (F(2, 62) = 4.45, *p* = 0.014, η^2^ = 0.06). Multiple comparison tests (with the Bonferroni adjustment) showed that for the low WMC group, the instructor’s presence (either in the format of a face picture (VP) or a talking head (VV)) led to a better learning performance (*p*s < 0.05), while for those with a high WMC, the videos with or without the instructor’s presence resulted in the same comprehension performance (*p*s > 0.05). Moreover, the high WMC group achieved significantly higher comprehension scores than the low WMC group in the instructor-absent video lectures (VN) (*p* < 0.01). In the instructor-present conditions (VP and VV), the two groups showed a similar comprehension performance. 

### 3.2. Visual Attention Allocation of Learners with High and Low WMC

We conducted a series of independent samples t-tests to examine whether learners with different WMCs might differ in their attention distribution in each video type condition (Kokoç et al. 2020).

The VN condition without the instructor’s presence involved only two AOIs: the text and the topic-related picture. Independent samples t-tests revealed no statistically significant differences in the fixation count and dwell time percentage on both AOIs between the high and low WMC groups (*p*s > 0.05). Regardless of their WMC, all participants distributed their attention similarly when there was no instructor in the video lecture: more than 86% of their time was allocated to the text while watching the instructor-absent videos (see Table 2).

The VP condition with the instructor’s face image included three AOIs: the text, the topic-related picture, and the instructor’s image. The results showed that both groups had similar attention allocation on the text AOI and the picture AOI, their fixation counts and dwell time percentages in these two areas. However, for the instructor AOI, the high and low WMC groups significantly differ, indicated by their attention allocation. Specifically, participants with a low WMC spent more time on the instructor’s image compared to those with a high WMC, as indicated by their significantly different fixation count percentages (*p* < 0.05) (Table 2) and marginally significantly different dwell time percentages (*p* = 0.058) (Table 2). Finally, we also examined the number of transitions between the instructor area and the other two AOIs. No significant differences in this metric were found between these two groups. 

The VV condition included a synchronized video of the instructor introducing the content and comprised the same AOIs as the VP condition: the text, the topic-related picture, and the instructor. Similarly to the VP condition, the independent samples t-tests showed that the two groups did not differ in their attention allocation in the text AOI and the picture AOI. However, they had different time allocation patterns on the instructor AOI. To be more specific, participants with a low WMC spent more time viewing the instructor than those with a high WMC, as indicated by their significantly greater fixation count percentage (*p* < 0.05) (Table 2) and dwell time percentage (*p* < 0.05) (Table 2). In addition, similarly to the VP condition, no significant differences were found in the number of transitions between fixating on the instructor and other contents between these two groups.

## 4. Discussion

Learners have a limited WMC, which assists in generative, extraneous, and essential multimedia learning processing. The instructor’s presence as a social cue in video lectures may facilitate the generative processing. It may also induce extraneous processing that overloads the learner’s WMC and impairs their learning. The present eye-tracking study explored the modulation of the learners’ WMC on the instructor presence effect in video lectures. We found that learners with a low WMC, compared to those with a high WMC, spent more time fixating on the onscreen instructor and benefited more from the instructor’s presence. The two groups were significantly different in their learning performance of the instructor-absent videos. However, with an instructor’s face picture (the VP condition) or talking head (the VV condition), the low-WMC participants caught up with their high-WMC peers. Our findings provide empirical evidence for the instructor presence effect and suggest that the instructor’s presence in video lectures is vital for low WMC learners.

### 4.1. The Beneficial Effect of Instructor Presence: Only for Learners with a Low WMC

In the present study, learners watched video lectures on unfamiliar topics, which were not difficult based on their self-reports. The learning outcome indicated by the comprehension scores revealed that adding an instructor to the video lectures was advantageous. As hypothesized, the individual differences in WMC modulate the instructor presence effect. However, contrary to our prediction, the onscreen instructor (in the VP and VV conditions) reinforced the comprehension performance of the low WMC group but not the high WMPC group. Eye-tracking data analyses revealed that the low WMC group focused significantly more on the instructor when viewing instructor-present videos than the high WMC group.

According to the social agency theory (Mayer 2014), the onscreen instructor as a social cue enhances the learners’ feelings of social presence and promotes generative processing. Thus, learners actively integrate learning content with their existing knowledge in the presence of an instructor. In the current study, the high WMC group had a significantly better learning performance than the low WMC only when viewing video lectures without an instructor. Participants with a high WMC had a similar comprehension performance in the three video types (VN, VP, VV). Therefore, in the case of the high WMC group, neither the instructor’s picture nor the instructor’s video constituted an aid or a distraction for their video lecture learning. That is to say, the presence of the instructor had a minimal and negligible influence on the generative and extraneous processes of the learners with a high WMC. Therefore, the influence of the instructor’s presence on their behavioral performance was insignificant. That said, it is possible that the instructor’s presence could modulate the learning performance of high-WMC learners, which might be evident measured by neurobiological techniques (e.g., event-related potentials, functional magnetic resonance imaging). It is also important to note that the current study didn’t examine other learning outcomes, such as affective arousal (e.g., Wang and Antonenko 2017; Wang et al. 2020) and learning transfer (e.g., Colliot and Jamet 2018; Pi and Hong 2016; Pi et al. 2020). 

For learners with a low WMC, the onscreen instructor seemed to be a conducive tool for understanding video lectures. Low WMC learners performed significantly worse than the high WMC group in the instructor-absent videos. Such group differences, however, disappeared in the instructor-present videos. In other words, even though the instructor is only displayed as a static face image on the screen (i.e., in the VP condition), people with a low WMC were more sensitive to their instructor’s presence and profited more from it. Despite having a low WMC, they were not overburdened by the instructor’s presence. Thus, contrary to what we had predicted, the low WMC group was facilitated, but not hindered, by the presence of the instructor in video lectures. Interestingly, we found that the instructor’s neutral face image as a low-embodied social cue facilitated learning in our low WMC group, which is consistent with a recent study by Wang et al. (2018). They found that students performed better in the transfer test when there was an image of an onscreen agent compared to the instructor-absent condition. Although Mayer (2021) admitted that their studies on the instructor image did not produce strong and consistent support for the instructor presence effect, the effect size in their investigations favored the format of having the instructor’s image on the screen during online lectures. 

In sum, our findings support the facilitative function of an instructor’s presence on learning performance (Chen and Wu 2015; Colliot and Jamet 2018; Pi and Hong 2016; Van Gog et al. 2014; Wang and Antonenko 2017; Zhang and Yang 2022) and have provided further empirical support for the social agency theory (Mayer 2014). The instructor’s presence did not induce extraneous processing in both groups when they viewed video lectures. While the high WMC group did not need the social cues to learn deeply, the low WMC learners made the best out of the instructor’s presence and performed similarly to those with a high WMC in the instructor-present video lectures. 

### 4.2. Learners with Low WMC Pay More Attention to the Instructor in Video Lectures

As we hypothesized, the presented instructor attracted more attention from the low WMC group, as evidenced by more fixations and a longer dwell time on the instructor AOI compared to the high WMC group (Table 2). Researchers suggest that learners with different WMCs differ mainly in their ability to control attention (Baddeley 1993; Engle et al. 1999; Mashburn et al. 2020; Unsworth et al. 2021; Wiley et al. 2014). Our high-WMC group paid less attention to the instructor and focused more on the learning content than the low-WMC group (see Table 2). Attention-grabbing extraneous materials (e.g., the instructor’s face in the current study) could distract low-WMC learners and increase their cognitive load (Sweller et al. 2011), which hinders learning. Nevertheless, none of the learners in the current study experienced any distracting effects from the instructor’s presence. Instead, as already mentioned, students seemed to benefit from the instructor’s presence.

Our results also demonstrated that students paid greater attention to the instructor’s talking head (in the VV condition) than to the instructor’s face image (in the VP condition), regardless of their WMCs. The high and low WMC groups gave the instructor’s face image 2.5% and 3.71% of their attention in video lectures. When the instructor was presented in the form of a talking head (VV), these percentages rocketed to 9.08% and 14.66% (see Table 2). Compared with the instructor’s face image, the instructor’s talking head provided more social cues, such as eye movements, mouth movements, facial expressions, and hand gestures. Those non-verbal cues may account for the on-screen instructor’s beneficial role to the low-WMC learners.

### 4.3. The Onscreen Instructor for Learners with Low WMC: Not a Distraction but an Aid

In the current study, the low WMC group was the only one to experience the instructor presence effect. For them, the additional onscreen instructor (either a talking head or simply just a face image) was not distracting but retained its positive value as a social cue. That is to say, the supposedly disadvantaged group (learners with a low WMC) managed to reap the instructor-presence benefits in video lectures. Our results align with earlier studies on learners’ prior knowledge. Mayer (2021) contended that many multimedia design principles are subjected to learners’ prior knowledge. Learners with low prior knowledge need additional guidance and support to build connections between pictorial and verbal representations (Mayer 2021). In our study, the low WMC group received extra support from the instructor’s presence, and they benefited more from this multimedia design than high WMC learners. Additionally, our results align well with recent research on the interaction between digital technology and L2 learners. Technology-enhanced multimodal information may affect learners with different cognitive capacities and aptitudes (see Li et al. 2020 for a recent review). For example, Legault et al. (2019) discovered that struggling L2 learners benefited more from the virtual reality (VR) platform than successful learners.

The poor WMC group’s comprehension performance was unaffected by the attention paid to the instructor on the screen. The videos in the current study might not be challenging even for low-WMC learners. They might not have an overburdened cognitive load in this low-load learning context. They subconsciously took advantage of the instructor’s onscreen presence as a valuable social cue to compensate for their inadequate learning ability in video learning. Our findings support this proposal by demonstrating that appealing details promote learning in low-load situations (Park et al. 2011).

Finally, our findings provide insights for future research into the underlying mechanisms of instructor-present video learning. For instance, the instructor’s presence provides social cues (e.g., eye gazes) and linguistic cues (from the talking mouth or head). Thus, which of these cues is more helpful for learning, particularly for those with low cognitive abilities? Is the social presence offered by the social cues (e.g., eye contact, facial expression) or the more direct assistance from the linguistic cues (e.g., such as lip movements) more beneficial? The cognitive process behind the instructor presence effect in low WMC learners may be clarified further in subsequent studies using more advanced instructional designs or techniques.

### 4.4. Implications, Limitations, and Future Research

To our knowledge, this is the first study to investigate how learning from instructor-presented video lectures is related to learner variations in WMC. Our results showed that learners’ WMC moderated the instructor presence effect. In this study, learners with a low WMC benefited more from the addition of an instructor to the video lecture than did learners with high cognitive capacities. The results offer evidence against the claim that learners are distracted by the onscreen instructor. Even those students who were thought to be prone to distractions in this study did not experience any detrimental effects from paying attention to the presented instructor. The teacher should, therefore, be visible on the video screen to promote social presence, which is advantageous for all learners in general and those with a low WMC in particular. This is the direct practical implication for video lecture designers. The results of the current study further highlight the importance of evaluating the differential effects of learners’ cognitively based individual characteristics on their learning outcomes and attention allocation in online video lectures. Further research is still needed to determine which learner types will gain the most from watching the onscreen instructor.

Although the study added new evidence for the benefits of a teacher’s presence in video lectures, it is crucial to note that our findings need to be interpreted with some caution. First, in the current study, we adopted a within-subjects design. As pointed out by an anonymous reviewer, though the order of video presentation was randomized, it was unlikely to completely eliminate the potential carry-over effects. Future research can address this problem by adopting a different approach, such as a between-subjects design with matched participants to further investigate the issue. Second, we assessed participants’ prior knowledge of each video topic using a five-point Likert scale rather than a more objective prior knowledge test. Third, participants self-reported that each video was easy and moderate to comprehend. Each video’s low-load context may make it less likely to tax students’ meager attentional resources. Future research could investigate whether an instructor’s presence in a high-load situation causes varying magnitudes of the instruction presence effect in learners with various WMCs. The difficulty of the topics could be included as a moderating variable. Finally, the study’s comprehension check exam was a straightforward retention test. As Mayer (2021) stated, it could be preferable to include a transfer test to determine how well learners grasp the content rather than only asking how much information has been remembered (as tested by the retention test). Future studies should thus include transfer tests to investigate whether learners with different WMCs will transfer their knowledge from the instructor-presented videos differently.

## 5. Conclusions

The current study explored the influence of learners’ WMC on their learning performance in video lectures with an instructor either present or absent. We found that the presence of an instructor, particularly the talking head of the instructor, was beneficial for video lecture learning, particularly for learners with a low WMC. Future research should examine learners with various WMCs in high-load video lectures and examine diverse learning outcomes with neurological techniques.

## Figures and Tables

**Table 1:** Average comprehension scores of the three video types in the high and low WMC groups.

Video Type	High WMC	Low WMC
VN	20.78 (1.60)	19.38 (2.09)
VP	20.47 (2.03)	20.47 (1.76)
VV	21.25 (1.93)	21.22 (1.77)

Note: VN, video lectures with an instructor’s voiceover but without presence; VP, video lectures with the instructor’s face picture; VV, video lectures with the same instructor talking. High WMC, the group with high working memory capacity; Low WMC, the group with low working memory capacity. The maximum score for each condition was 24.

**Table 2:** Visual attention distribution statistics for the three types of video lectures.

AOI	Measure	VN		VP		VV	
		High WMC	Low WMC	High WMC	Low WMC	High WMC	Low WMC
Text	Fixation count (%)	87.89 (5.13)	87.14 (5.88)	81.26 (6.67)	79.89 (7.63)	80.10 (8.34)	77.42 (10.23)
	Dwell time (%)	87.29 (6.03)	86.44 (7.53)	80.68 (7.80)	79.66 (8.99)	77.71 (10.78)	73.15 (13.55)
Picture	Fixation count (%)	10.75 (4.74)	11.12 (5.05)	14.45 (6.43)	14.07 (5.87)	11.39 (6.50)	10.25 (4.80)
	Dwell time (%)	11.57 (5.69)	12.26 (6.73)	15.54 (7.71)	15.08 (7.40)	11.91 (7.52)	10.99 (6.13)
Instructor	Fixation count (%)			2.57 (2.13)	3.85 (2.48) *	6.77 (5.58)	10.56 (7.57) *
	Dwell time (%)			2.50 (2.32)	3.71 (2.67)	9.08 (7.39)	14.66 (11.48) *
	Number of transitions ^a^			7.43 (6.57)	9.25 (6.45)	14.59 (12.13)	19.91 (15.17)

Note: AOI, area of interest. VN, video lectures with an instructor’s voiceover but without presence; VP, video lectures with the instructor’s face picture; VV, video lectures with the same instructor talking. High WMC, the group with high working memory capacity; Low WMC, the group with low working memory capacity. ^a^ Average number of times the instructor AOI was entered and left; * indicated a statistically significant difference between the high and low WMC groups.

## Data Availability

Not applicable.

## Note

1 Part of the data was reported in a paper on gender differences in learning via video lectures (Zhang and Yang 2022).

## Supplementary Materials

The following supporting information can be downloaded at: https://www.mdpi.com/article/10.3390/jintelligence11010005/s1, Table S1: Descriptives of 11 lecture videos.
