# Supplementary material for: Learners with Low Working Memory Capacity Benefit More from the Presence of an Instructor’s Face in Video Lectures
Source: J Intell. 2022 Dec 27;11(1):5. doi: 10.3390/jintelligence11010005 (PMC9861543; doi:10.3390/jintelligence11010005)
Supplement: Supplementary file 1 [file jintelligence-11-00005-s001.zip › jintelligence-1991371-supplementary.pdf]

Table S1. Descriptives of 11 lecture videos.

NO.	Topic	Area	Video type	Familiarity [a]	Difficulty [a]	Familiarity [b]	Difficulty [b]
1*	Venus	science	PV	3.21	2.47	2.29	2.70
2*	Volcano	science	VV	3.63	2.47	2.92	2.65
3	Rosetta stone	history	VV	1.37	2.16	1.38	2.94
4	Medici	history	PV	1.95	2.05	1.91	2.18
5	Copper age	history	AV	1.58	2.63	1.56	2.59
6	The sound and the fury	literature	PV	2.42	2.26	1.56	3.06
7	Isabel Allende	literature	AV	1.42	2.05	1.58	2.41
8	Malin Kundang [c]	literature	VV	1.26	1.58	1.42	2.00
9	Rhizanthella gardneri [d]	science	PV	1.31	2.15	1.50	2.56
10	Balinese tiger	science	AV	1.95	1.79	1.76	2.30
11	Permafrost	science	VV	2.16	2.42	2.53	2.67

AV: the audio-video condition; PV: the picture-video condition; VV: the video-video condition; * used as practice; [a] rated by 20 Chinese students who did not participate in the study; [b] rated by the 64 participants in the eye-tracking experiment; [c] a folk tale in Southeast Asia; [d] an entirely subterranean mycoheterotrophic orchid.
